# Circulating extracellular vesicle microRNAs associated with adverse reactions, proinflammatory cytokine, and antibody production after COVID-19 vaccination

**DOI:** 10.1038/s41541-022-00439-3

**Published:** 2022-02-08

**Authors:** Yusuke Miyashita, Takanobu Yoshida, Yuriko Takagi, Hirotake Tsukamoto, Ken Takashima, Takahisa Kouwaki, Katsunari Makino, Satoshi Fukushima, Kimitoshi Nakamura, Hiroyuki Oshiumi

**Affiliations:** 1 Department of Immunology, Graduate School of Medical Sciences, Faculty of Life Sciences, Kumamoto University, 1-1-1 Honjo, Kumamoto, 860-8556 Japan; 2 Department of Pediatrics, Graduate School of Medical Sciences, Kumamoto University, 1-1-1 Honjo, Kumamoto, 860-8556 Japan; 3 Division of Clinical Immunology and Cancer Immunotherapy, Center for Cancer Immunotherapy and Immunobiology, Graduate School of Medicine, Kyoto University, Kyoto, Japan; 4 Department of Dermatology and Plastic Surgery, Faculty of Life Sciences, Kumamoto University, 1-1-1 Honjo, Kumamoto, 860-8556 Japan

**Keywords:** Adjuvants, RNA vaccines

## Abstract

mRNA-based vaccines have been used globally to eradicate the coronavirus-disease 2019 (COVID-19) pandemic. Vaccine efficacy and adverse reactions depend on immune responses, such as proinflammatory cytokine production and lymphocyte activation. We conducted a prospective cohort study to investigate relationships among specific antibody titers, adverse reactions, proinflammatory cytokine production, and immune-regulatory microRNA (miRNA) levels in serum extracellular vesicles (EVs) after COVID-19 vaccination (BNT162b2). Local adverse reactions after the second dose, such as local pain and swelling, were less correlated with those of systemic symptoms, such as fever and muscle pain, whereas serum TNF-α levels were associated with systemic adverse reactions and with specific antibody titers. Interestingly, EV miR-92a-2-5p levels in sera were negatively correlated with degrees of adverse reactions, and EV miR-148a levels were associated with specific antibody titers. Our data suggest a potential of circulating EV miRNAs as biomarkers for vaccine efficacy and adverse reactions.

## Introduction

Vaccination is the best prophylaxis to prevent infectious diseases, and vaccines comprise antigens and adjuvants. Adjuvants elicit innate immune responses, such as proinflammatory cytokine production and dendritic cell maturation, required for activating adaptive immune responses, leading to antigen-specific antibody production^1^. Aluminum salts have been used as adjuvants in many types of vaccines^2–4^, and ligands for pattern-recognition receptors, such as toll-like receptors, are also used as adjuvants^5^. However, even in the absence of those molecules, components of the vaccine itself could function as adjuvants. For instance, viral RNAs within the whole-virus vaccine of influenza-A virus activate TLR7, thereby evoking innate immune responses, including proinflammatory cytokine production^6^.

Although vaccine-induced adverse reaction itself is not serious in many cases, fears of adverse reactions sometimes drop the vaccination rate, resulting in insufficient eradication of infectious diseases, such as human papillomavirus-induced cervical cancers^7^. Thus, appropriate understanding of the mechanism underlying adverse reactions is important to dispel the fear. A well-known adverse reaction is anaphylaxis, which is a type-I hypersensitivity, with a sufficiently established mechanism and treatment regimen^8,9^. Other adverse reactions, such as fever, local pain, swelling, and redness, are supposed to be caused by proinflammatory cytokine and lipid mediators^10–14^. Moreover, it has been shown that systemic proinflammatory cytokine is a cause of those adverse reactions after seasonal flu vaccination^15^.

Extracellular vesicles (EVs), including exosomes and microvesicles, are enriched in the blood, and contain functional proteins and RNAs, delivering them from donor to recipient cells^16,17^. MicroRNAs (miRNAs) target mRNAs and regulate their translation^18–20^. EVs contain immune-regulatory miRNAs, and EV miRNAs have been shown to regulate host innate and adaptive immune responses^21^. Previously, we found that miR-451a levels in serum EVs were weakly associated with adverse reactions after seasonal flu vaccination^14,22^.

SARS-CoV-2 causes the coronavirus-disease 2019 (COVID-19) pandemic in 2020^23,24^, and several COVID-19 vaccines have been approved and used worldwide to eradicate the coronavirus disease. BNT162b2 (Pfizer) is a COVID-19 vaccine that contains mRNA-encoding viral spike proteins^25^. It has been reported that after vaccination with BNT162b2, there are several adverse reactions, such as local pain, redness, swelling, fever, fatigue, and muscle pain^25^, which are supposed to be caused by immune responses. However, it remains unknown whether these adverse reactions are associated with proinflammatory cytokines and EV miRNAs. Additionally, whether the viral spike-protein-specific antibody production is related to the severity of adverse reactions, proinflammatory cytokine, and miRNA levels is unclear. To investigate the correlations among these factors, we conducted a prospective cohort study with BNT162b2.

## Results

### Demographic characteristics and vaccination data

**Fig. 1:**
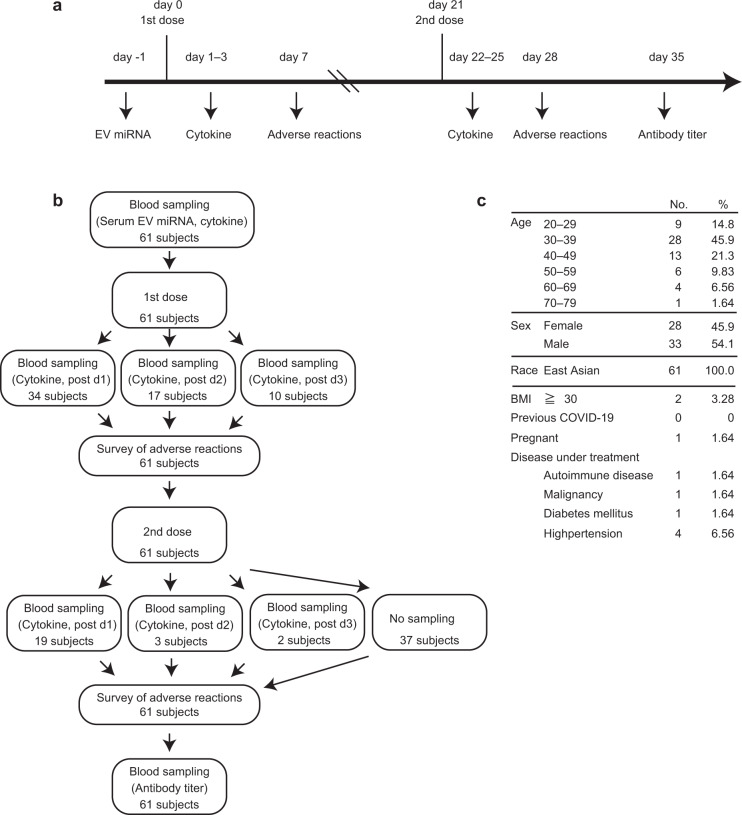
Enrollment and demographic characteristics of the participants. **a** Schematic of the schedule of sampling and vaccination. **b** The diagram represents all enrolled participants. The numbers of subjects at the indicated vaccination or sampling steps are shown. **c** Demographic characteristics of all participants.

Between March and June 2021, 61 persons were enrolled and received two doses of BNT162b2 as shown in Fig. 1a and b. The demographic characteristics of study samples are shown in Fig. 1c. The gender ratio is almost 1:1 (1.18 male to female), and all the participants are of Asian ethnicity, 45.9% of the population aged 30–39 years, and 18% of participants are more than 50 years old. All participants were healthy or had stable medical conditions and received two doses of BNT162b2 (Pfizer) (Fig. 1c). Sera were collected from 61 subjects before and after the first dose and from 24 subjects after the second dose (Fig. 1a and b). No one had severe adverse events such as death, hemorrhagic stroke, and myocardial infarction after vaccination.

### Local and systemic adverse reactions after COVID-19 vaccination

**Fig. 2:**
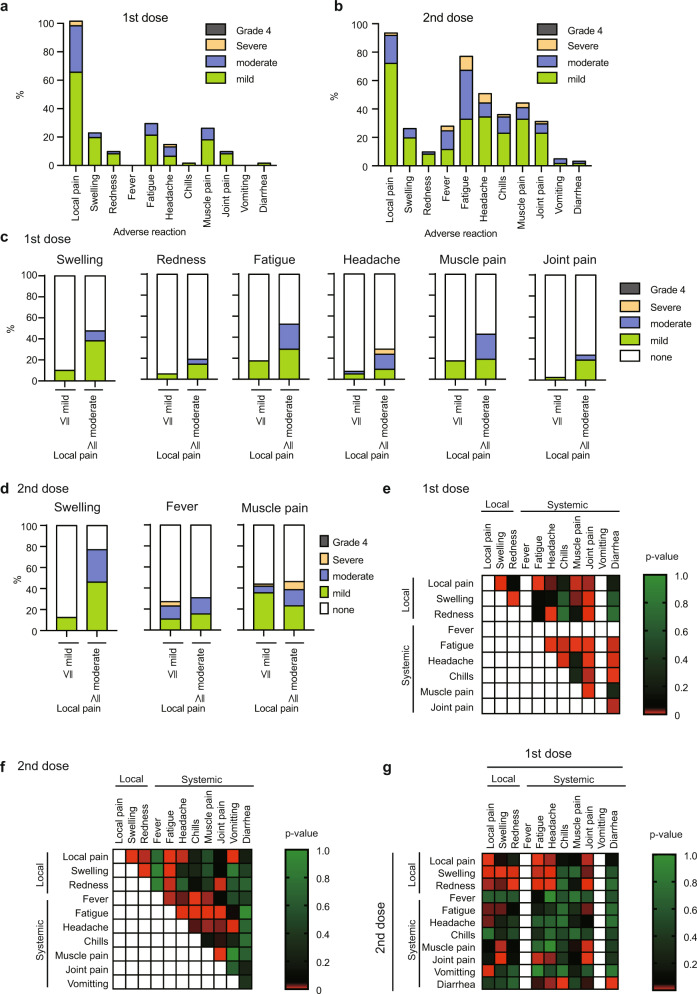
Adverse reactions after the first and second doses. **a**, **b** Data on local and systemic symptoms of 61 patients within 1 week after the first (**a**) and second (**b**) doses were collected as described in the “Methods”. The data are shown as percentages of the degree of each symptom (*n* = 61). **c**, **d** Subjects were classified into two groups, ≦mild (no or mild local pain) and ≧moderate (mild, severe, and grade-4 local pain), and percentages of each symptom degree are shown. **e**–**g** Degrees of each symptom were scored as no (0), mild (1), moderate (2), severe (3), and grade 4 (4), and *p*-values of the correlation coefficient of scores between two symptoms after the first dose (**e**), after the second dose (**f**), and after the first and second doses (**g**) were calculated. *P*-values are shown as heat maps. Red color represents statistical significance (*p* < 0.05).

First, we collected the degree of local and systemic symptoms from 61 subjects after the first and second doses, as indicated in Fig. 1. Almost all subjects had local pain at injection sites after the first and second doses. The degrees of other adverse reactions were more severe after the second dose than those after the first dose (Fig. 2a, b), which was consistent with a previous report^25^. Comparing the level of each symptom, all subjects were classified into two groups: one group has no or mild local pain, and the other has a more severe local-pain symptom. After the first dose, the group with severe local pain has more severe swelling, redness, fatigue, headache, muscle pain, and joint pain (Fig. 2c and Supplementary table 1). By contrast, the differences in the degree of fever or muscle pain after the second dose were relatively marginal between the two groups, although swelling was more severe in subjects with severe local pain (Fig. 2d and Supplementary table 1).

To further investigate the relationship among each symptom, the degree of each adverse reaction was scored from 0 to 4, according to the severity, such as none = 0, mild = 1, moderate = 2, severe = 3, and grade 4 = 4. We calculated the statistical significance (*p*-values) of correlations of each pair of adverse reactions. Additionally, local and systemic scores were defined as sums of scores of local and systemic symptoms, respectively.

Interestingly, a heatmap of the *p*-values of correlation of two symptoms suggested that scores of adverse reactions were roughly correlated with each other after the first dose (Fig. 2e). However, after the second dose, scores of local adverse reactions were not related to those of the systemic adverse reactions, such as fever, chills, muscle pain, and joint pain (Fig. 2f). By contrast, the systemic symptoms, such as fever, fatigue, and muscle pain, were correlated with each other after the second dose (Fig. 2f).

We also found that scores of the local symptoms were roughly correlated between the first and second doses (9 blocks on the upper left in Fig. 2g), but those of systemic symptoms were not (blocks on the lower right in Fig. 2g), implying that the systemic symptoms after the second dose are not associated with the local symptoms. To further elucidate this point, we compared local and systemic scores. The coefficient of determination between local and systemic scores after the second dose was below 0.2 (Supplementary Fig. 1). By contrast, the coefficient of determination between the local and systemic scores after the first dose was above 0.2 (R^2^ = 0.26), suggesting a weak correlation (Supplementary Fig. 1). These data also suggest that systemic symptoms after the second dose are not associated with local symptoms. Moreover, we found that coefficients of determination between local scores after the first and second doses were above 0.2 (R^2^ = 0.36) (Supplementary Fig. 1).

Subjects with antipyretic medication had more severe local and systemic symptoms than those without it (Supplementary Fig. 2), which is consistent with the assumption that subjects with severe symptoms would take an antipyretic medication.

### IL-6 and TNF-α levels in sera after vaccination

**Fig. 3:**
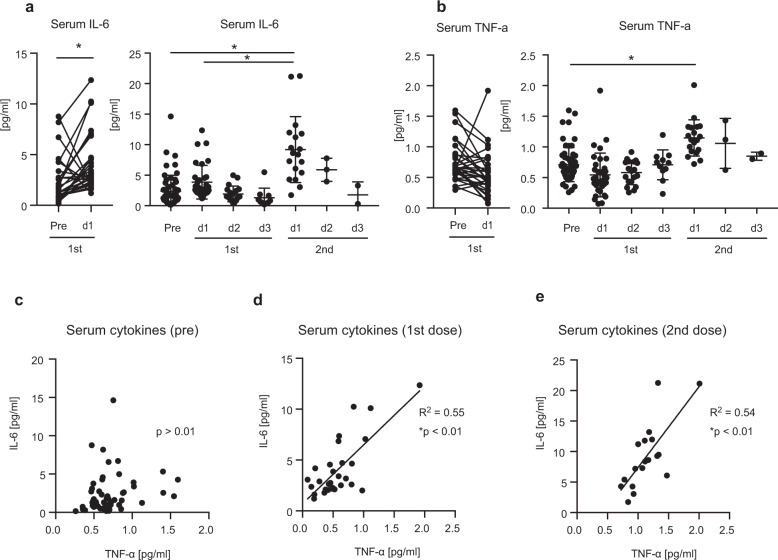
Serum IL-6 and TNF-α levels before and after vaccination. **a**, **b** Sera were collected before (pre) and after the first and second doses (days 1, 2, or 3). Serum IL-6 (**a**) and TNF-α (**b**) protein levels were determined by ELISA. Paired *t*-test (pre and post 1 day) and one-way ANOVA (each right panel) were performed (*p < 0.05). The bars represent mean ± standard deviation (SD). **c**–**e** Correlation between IL-6 and TNF-α protein levels at pre (**c**), 1 day after the first dose, and 1 day after the second dose (**e**) was investigated. The coefficient of determination (R^2^) and *p*-values were calculated (Pearson correlation test, *p < 0.05). Each dot represents a subject.

It is previously reported that the production of proinflammatory cytokines after vaccination is associated with adverse reactions^15,26^, and thus, we measured serum proinflammatory cytokine levels. IL-6 levels 1 day after the first dose were elevated compared with the levels before vaccination, and the levels were further elevated after the second dose (Fig. 3a). Serum TNF-α levels did not increase after the first dose but increased significantly after the second dose (Fig. 3b). Although there was no correlation between IL-6 and TNF-α levels before vaccination (Fig. 3c), there were moderate correlations between serum IL-6 and TNF-α levels after the first and second doses (Fig. 3d and e). These data suggest that proinflammatory cytokines were produced in response to BNT162b2 vaccination, especially after the second dose.

**Fig. 4:**
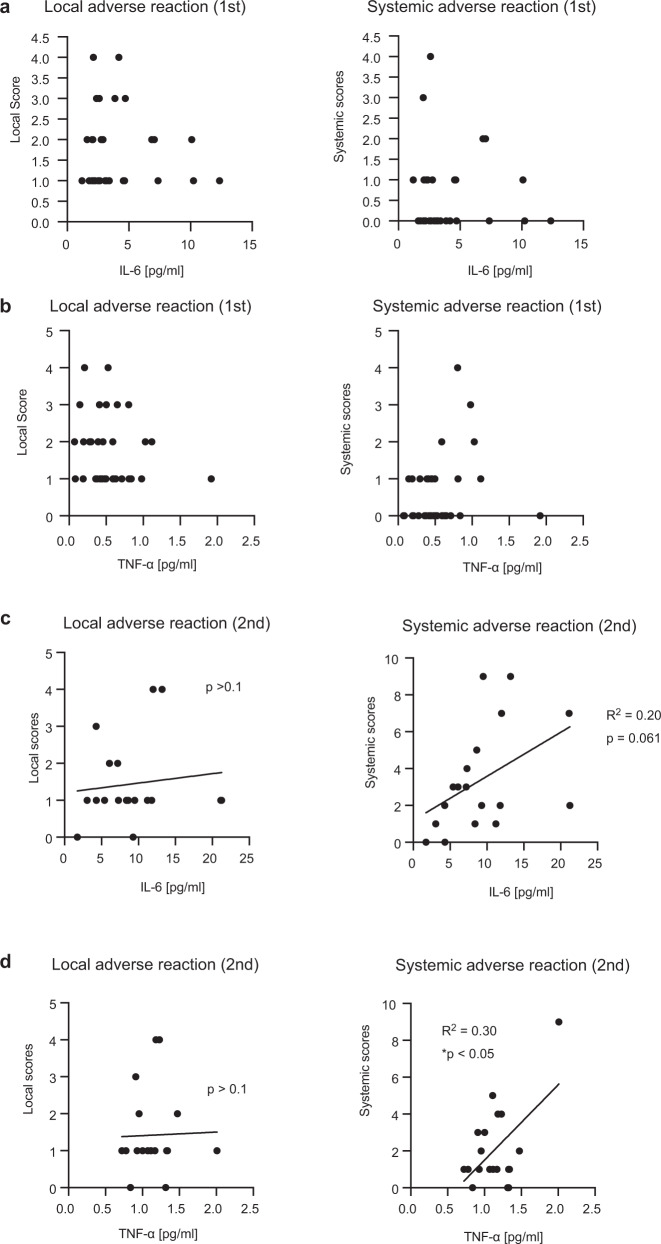
Correlation between cytokine level and degree of symptoms. **a**, **b** Local and systemic scores were compared with serum IL-6 (**a**) and TNF-α (**b**) levels 1 day after the first dose. There were no correlations. **c**, **d** Local and systemic scores were compared with serum IL-6 (**c**) and TNF-α (**d**) levels 1 day after the second dose. The coefficient of determination (R^2^) and *p*-values were calculated (Pearson correlation test, *p < 0.05). Each dot represents a subject.

Next, we investigated the correlation between proinflammatory cytokine levels and adverse reactions after vaccination. We chose serum samples collected at 1 day post vaccination for comparison, because cytokine levels exhibited a trend to decrease within a few days (Fig. 3). After the first dose, we could not observe any significant correlation between proinflammatory cytokine levels and adverse reaction scores (Fig. 4a and b). Although local scores after the second dose were not correlated with either IL-6 or TNF-α level in sera, systemic scores were correlated with serum TNF-α levels after the second dose (Fig. 4c and d). This is consistent with the notion that proinflammatory cytokine is a cause of adverse reactions after vaccination^26^.

### TNF-α in serum is correlated with the vaccine efficacy

**Fig. 5:**
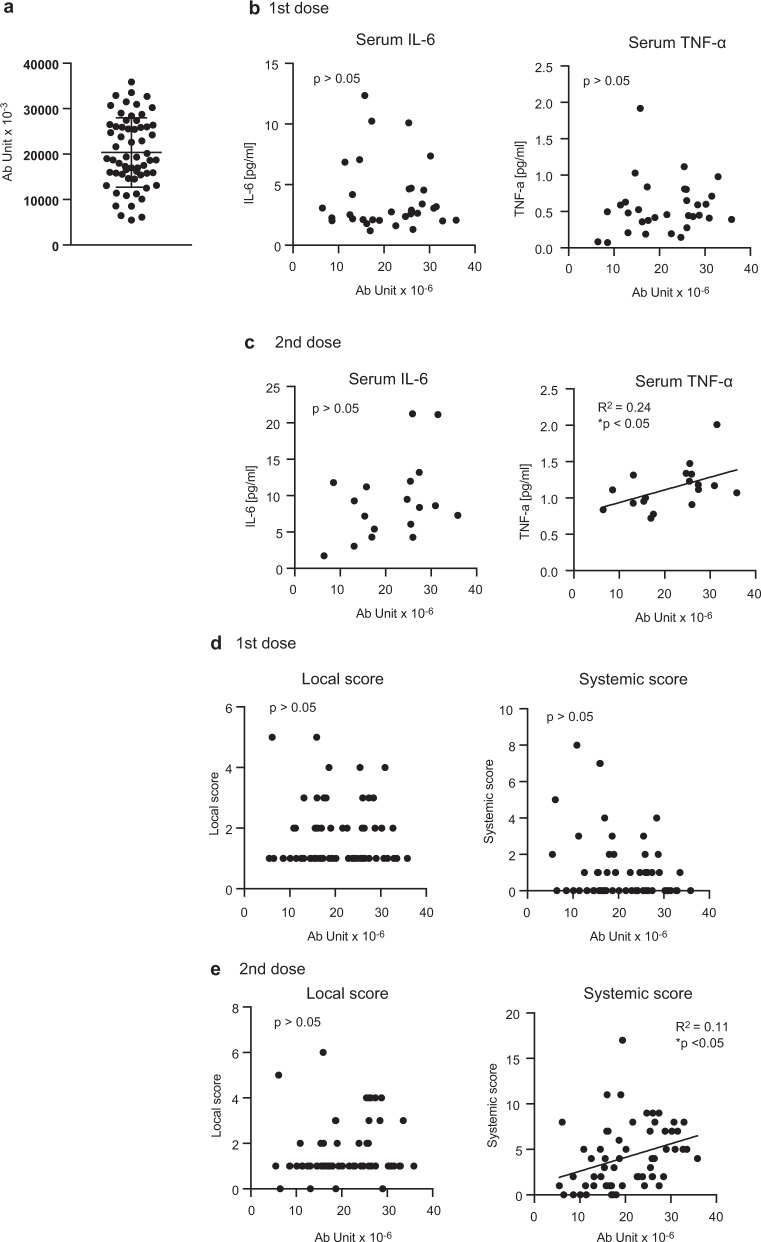
Anti-spike-protein antibody titers. **a** Anti-spike-protein antibody titers in sera at 35 days after the first dose were determined via ELISA. **b**, **c** Specific antibody titers were compared with serum IL-6 and TNF-α levels 1 day after the first (**b**) and second (**c**) doses. **d**, **e** Specific antibody titers were compared with local and systemic scores after the first (**d**) or second (**e**) doses. The coefficient of determination and *p*-values of correlation were calculated (Pearson correlation test, *p < 0.05). Each dot represents a subject.

Next, we determined SARS-CoV-2 spike-protein-specific antibody levels in sera (Fig. 5a), and investigated whether proinflammatory cytokine levels were associated with specific antibody levels. We confirmed that treatment with an antipyretic medication did not reduce specific antibody titers, but the titers were higher in the subjects with treatment than in those without it (Supplementary Fig. 3).

Serum IL-6 and TNF-α levels after the first dose were not correlated with S-protein-specific antibody titers (Fig. 5b), but serum TNF-α levels after the second dose were associated with specific antibody titers (Fig. 5c).

We next investigated the correlations between adverse reactions and specific antibody titers. Local scores after the first and second doses were not correlated with specific antibody titers (Fig. 5d and e), but systemic scores after the second dose were associated with specific antibody titers (Fig. 5e). These observations are consistent with the notion that adverse reactions and proinflammatory cytokine production are caused by immune responses to antigens, leading to production of antigen-specific antibody production.

### Circulating EV microRNAs associated with immune responses

**Fig. 6:**
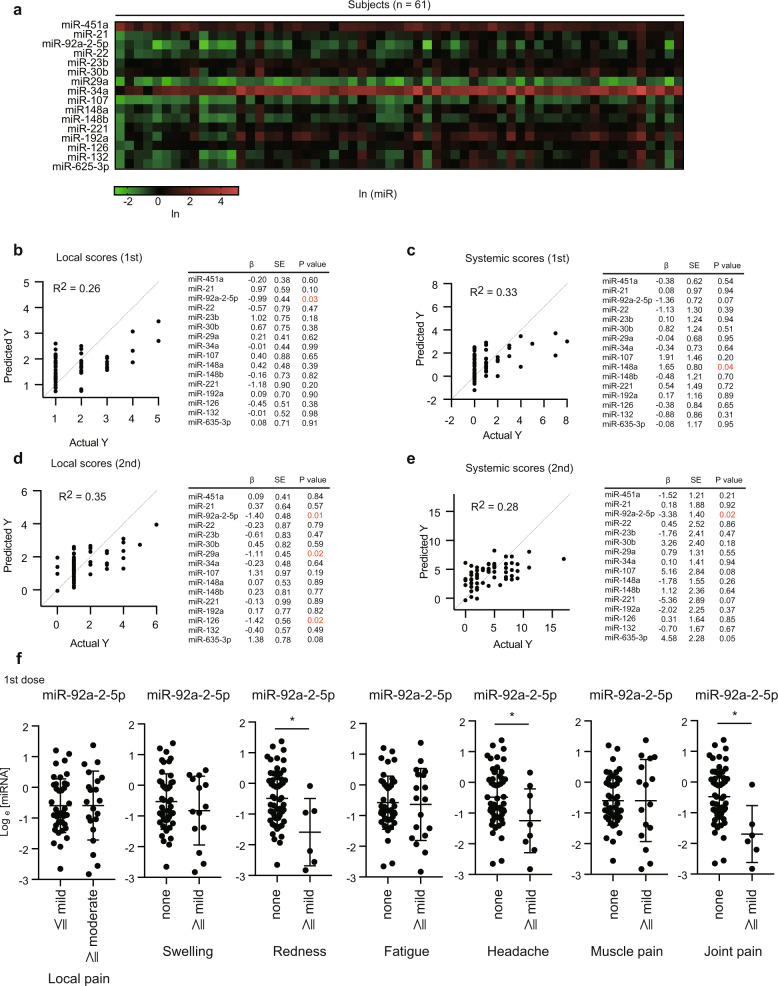
Serum extracellular-vesicle miRNA levels. **a** EV miRNA levels in sera collected at 1 day before the first dose were determined by RT-qPCR. The heatmap represents the ln (miRNA) values of each miRNA. **b**–**e** Local and systemic scores after the first (**b**, **c**) and second (**d**, **e**) doses were compared with ln (miR) levels. Multiple linear-regression analyses were performed, and multiple coefficients of determination (R^2^) were calculated. The tables show coefficient (β), standard error (SE), and *p*-values of each ln (miR). **f** All subjects were classified into two groups on the basis of the degree of each symptom as indicated. Ln (miR-92a-2-5p) levels in the two groups were compared. Student *t*-tests were performed (*p < 0.05). The bars represent mean ± SD. Each dot represents a subject.

Since we had previously found that serum EV miRNA levels before vaccination were associated with adverse reactions after seasonal flu vaccination^14^, we measured several miRNA levels in serum EVs by RT-qPCR and compared them with local and systemic scores. The size and concentration of collected serum EVs are shown in Supplementary Fig. 4a. On the basis of previous reports^14,27–31^, we focused on 16 immune-regulatory microRNAs (Fig. 6a). miRNA levels in serum EVs were normalized to miR-16 levels, because we have previously found that miR-16 is suitable to normalize miRNA levels in human serum EVs rather than U6 RNA^22^. We have also found that miRNA levels normalized to miR-16 were correlated to cytokine expression in response to components of influenza vaccines^14,22^. Additionally, we confirmed that U6/miR-16 ratio in EVs released from macrophage cell line was not affected by stimulation with poly I:C, dsRNA analog, suggesting that miR-16 levels in EVs are stable as U6 levels are (Supplementary Fig. 4b). Interestingly, multiple-regression analysis suggested that miR-92a-2-5p levels in serum EVs 1 day before the first dose were negatively correlated with local scores after the first and second doses, and also correlated with systemic scores after the second dose (Fig. 6b–e). Additionally, miR-148a levels were positively correlated with systemic scores after the first dose (Fig. 6c). Unlike seasonal flu vaccination, miR-451a levels were not connected with any scores (Fig. 6b–e). Since EV miR-92a-2-5p levels were correlated with local and systemic scores, we investigated which symptoms were associated with miR-92a-2a-5p. Our statistical analyses suggested that miR-92a-2-5p levels in serum EVs were significantly decreased in subjects who had redness, headache, or joint-pain symptoms (Fig. 6f). Systemic scores of females were higher than those of males, but there was no difference in miR-92a-2-5p levels between females and males (Supplementary Fig. 5a, b), suggesting that miR-92a-2-5p is not associated with gender difference.

**Fig. 7:**
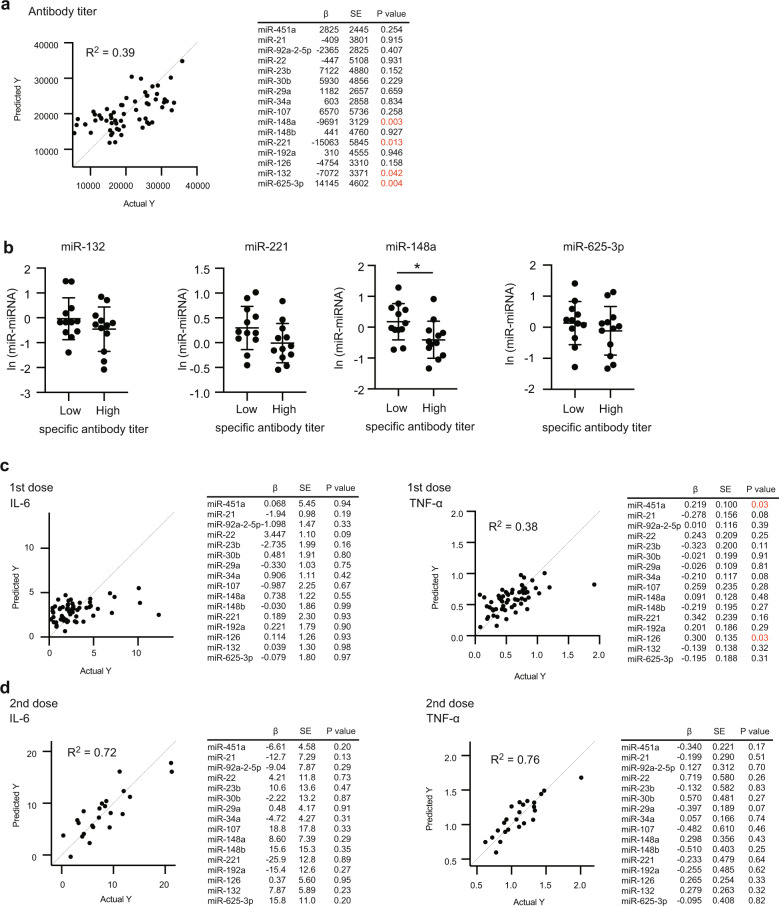
Correlation among miRNA, cytokine, and specific antibody levels. **a** Correlation between ln (miRNA) levels and specific antibody titers was investigated via multiple-regression analysis. Multiple coefficients of determination (R^2^) were calculated. The table shows the coefficient (β), standard error (SE), and *p*-values of each ln (miR). **b** ln (miR-132), ln (miR-148a), ln (miR-221), and ln (miR-625-3p) levels were compared between a group with a high specific antibody titer (top 20%) and that with a low level (bottom 20%). Each dot represents a subject (*p < 0.05, *t*-test). The bars represent mean ± SD. **c**, **d** Correlation between ln (miRNA) levels and cytokine levels 1 day after the first (**c**) or second (**d**) doses was investigated by multiple linear-regression analysis. Multiple coefficients of determination (R^2^) were calculated. The tables show coefficient (β), standard error (SE), and *p*-values of each ln (miR).

Subsequently, we compared miRNA levels with specific antibody titers. Multiple-regression analysis suggests the relationships of the level of miR-132, miR-148a, miR-221, and miR-625-3p with the antibody titers (Fig. 7a), whereas other miRNA levels were not connected with the antibody titers. To further investigate correlations between each miRNA and the antibody titers, we compared miRNA levels between the groups with high and low specific antibody titers. Interestingly, we found that miR-148a levels were significantly lower in the group with high antibody titers (Fig. 7b). Next, we performed multiple-regression analysis to compare the cytokine levels with serum EV miRNAs. miR-126 and miR-451 were connected to TNF-α levels after the first dose (Fig. 7c). Since TNF-α was not induced after the first dose, it is expected that TNF-α levels after the first dose reflect only basal TNF-α levels. No miRNAs were connected to TNF-α and IL-6 levels after the second dose (Fig. 7d). Collectively, our data imply that miR-92a-2-5p and miR-148a are expected to be associated with immune responses after vaccination with BNT162b2.

## Discussion

Adverse reactions of vaccines are thought to be caused by immune responses, and previous studies have shown that proinflammatory cytokine production is a cause of those adverse reactions^10–13^. This study investigated the correlation between proinflammatory cytokine levels in sera and adverse reactions after COVID-19 vaccination, and found that systemic TNF-α levels were connected with the systemic scores after the second dose. This observation also supports the notion that proinflammatory cytokine is a cause of adverse reactions after vaccination. Although we could not find significant correlation between the cytokine levels and local adverse reactions, it is possible that cytokine levels at the injection site were different from those in sera. Another possibility is that other cytokines and lipid mediators, such as prostaglandins, caused local symptoms, such as local pain, swelling, and redness at the vaccination site. However, further analyses must reveal the underlying mechanisms of adverse reactions.

Interestingly, the degrees of systemic symptoms were largely unrelated to local symptoms after the second dose. These data imply that factors causing local adverse reactions would be different from those causing systemic symptoms. Since innate immunity is expected to be activated in response to the components of vaccines at the vaccination site, local symptoms after the first and second doses might reflect the innate immune response, and thus, systemic symptoms after the second doses are expected to be caused by an adaptive immune response, such as T-cell-mediated proinflammatory cytokines.

Our study showed a correlation of specific antibody titers with serum TNF-α levels after the second dose. TNF-α has been reported to be required for the formation of primary B-cell follicles and essential for the production of antigen-specific IgG^32^. Recombinant TNF-α can also promote B-cell proliferation^33^. TNF-α is secreted from myeloid cells and B- and T cells in response to antigens^34^. Hence, TNF-α is expected to be secreted from lymphocytes in response to vaccination, resulting in augmented production of specific antibodies against the viral spike protein.

Previous studies have shown that circulating EV miRNAs regulate immune responses after vaccination. We have reported that miR-451 and miR-192 in EVs regulate proinflammatory cytokine expression in response to seasonal flu vaccine^14,22,27^. Unlike our expectation, we could not find any association of these miRNAs with local and systemic symptoms in this cohort study. It is possible that miR-451a and miR-192 might not be involved in immune responses caused by vaccination with BNT162b2. By contrast, we found that miR-92a-2-5p levels were negatively correlated with local and systemic scores, and miR-148a was associated with production of specific antibodies. These data suggest that miR-92a-2-5p and miR-148a are involved in immune responses to components of BNT162b2.

miR-92a-2-5p has been identified as a biomarker for small-cell lung cancer^35,36^, and later studies suggested that it targeted TLR2 and suppresses TLR-2-mediated liver fibrosis^37^. Our previous microarray analysis showed that miR-92a-2-5p was a miRNA highly expressed in serum EVs of subjects^14^. Although the role of miR-92a-2-5p in the immune response after vaccination remains unclear, we prefer the interpretation that miR-92a-2-5p might reflect some unknown physical condition related to immune responses. Furthermore, miR-148a was associated with specific antibody production. miR-148a negatively regulates innate immune response and antigen presentation by dendritic cells^38^. Considering that dendritic cells play crucial roles in priming naive T cells required for B-cell activation, leading to antibody production, it is expected that miR-148a would regulate dendritic cell function, thereby affecting the levels of specific antibodies after vaccination.

Interestingly, miR-126, miR-132, miR-221, and miR-625-3p were also connected with the antibody titers or serum-cytokine levels after vaccination in our regression analyses. miR-126 inhibits VCAM-1 expression in endothelial cells, thereby reducing leukocyte adherence to endothelial cells^29^, and miR-126 is known to be increased in sepsis^39^. miR-126-5p, which is produced from the same pre-miR-126 RNA as miR-126 is, binds to capsase-3 and prevents dimerization of the caspase^40^. Thus, miR-126-5p inhibits the caspase activity to limit apoptosis in endothelial cells^40^. Because endothelial cells are a source of EVs^41^, it is interesting to investigate whether miR-126-5p is concomitantly expressed with miR-126 and whether miR-126 and miR-126-5p affect the production of miRNA-containing EVs and immune responses after vaccination. miR-132 targets SOX4 in B cells required for B-cell development^30^, and miR-221 regulates redirection of precursor B cells to bone marrow^42^. But, it is still unclear whether these miRNAs are involved in immune responses after vaccination. Recent studies elucidated miRNAs related to SARS-CoV-2^43–45^. Functional analyses of these miRNAs are important to reveal the role of the miRNAs in immune responses after vaccination.

EVs include exosomes, microvesicles (also called ectosomes), and apoptotic bodies. Recent studies have shown that not only exosomes but also other vesicles deliver miRNAs to other cells^18–20^. Our nanoparticle-tracking analysis showed that most of isolated particles were smaller than 100 nm, although a part of particles was larger than 100 nm. It is possible that specific types of EVs contain miRNAs related to immune responses. Further studies are required to fully reveal the function of EVs in immune responses after vaccination.

In this study, we found several immune-regulatory miRNAs associated with a part of immune responses after COVID-19, including antibody titers and adverse reactions. miRNAs have been identified as biomarkers for several diseases. Alternatively, miRNAs in EVs have been reported to regulate immune responses. Most of the adverse reactions are not serious, however, fears against adverse reactions drop vaccination rate, and thus prevent herd immunity. Further studies on miRNAs are expected to identify new biomarkers to predict preventive effects and adverse reactions of vaccines, leading to an understanding of the mechanism underlying individual differences in the responses after vaccination.

## Methods

### Participants

Between March 2021 and June 2021, 61 participants, mainly including the staff and faculty members in Kumamoto University and its hospital, were recruited. Those with unstable-disease conditions or symptoms were not recruited to this study. All participants provided written informed consent. In total, 24 subjects agreed with the blood sampling after the second dose. Antipyretic medicines (Tylenol A) were handed to all participants, and they took the medicines if it was needed. All participants gave written informed consent prior to participation. The ethics committee of the Faculty of Life Science at Kumamoto University approved this study (RINRI 1524), and all experiments have been conducted according to the principles expressed in the Declaration of Helsinki.

### EV isolation and microRNA qPCR

According to the manufacturer’s protocol, EVs in serum were collected using a Total Exosome Isolation Kit (from serum, Thermo Fisher). Total RNA in EVs was extracted using the TRI reagent (MOR). miRNAs were reverse-transcribed using MirX miRNA First-Standard Synthesis Kit (Clontech). Quantitative PCR was conducted using Power SYBR Green Master Mix (Thermo Fisher) on Step One Real-Time PCR System (ABI). The levels of each miRNA are measured twice and its value was averaged. Expression of each miRNA was normalized to miR-16 expression using the comparative 2[−ΔΔC_t_] method. Primer sequences used for quantitative PCR analyses were listed in Supplementary Table 2.

### Vaccine data and the scores of subjective symptoms

Participants were vaccinated with two 30 μg doses of BNT162b2 (Pfizer), containing nucleoside-modified RNA encoding the SARS-CoV-2 full-length spike, apart from 21 days, except for two people (they are 34 days apart). Subjective symptoms were counted on the basis of a questionnaire completed by the subjects within 7 days of each vaccination. The questionnaire inquired about the degrees of each symptom, such as local pain, swelling and redness at the injection site or fever, fatigue, headache, chills, muscle pain, joint pain, vomiting, and diarrhea. Degrees of symptoms were classified according to a previous report^25^, and were scored as follows: none, 0; mild, 1; moderate, 2; severe, 3; and grade 4, 4. Local scores were determined as the sum of scores of local pain, swelling, and redness. Systemic scores were defined as the sum of scores of the degrees of fever, headache, chills, muscle pain, joint pain, vomiting, and diarrhea.

### ELISA

The levels of IL-6 and TNF-α in serum were analyzed at before vaccination, and post day 1, day 2, or day 3 after vaccination using a Quantikine HS ELISA human IL-6 (HS600C) or TNF-a (HSTA00E) immunoassay kit (R & D system) according to the manufacturer’s protocol. The levels of antibody against SARS-CoV-2 spike protein were analyzed at 14 days after the second dose using Human SARS-CoV-2 Spike (Trimer) Ig Total ELISA kit (Thermo Fisher) according to the manufacturer’s protocol. A few subjects exhibited too high basal IL-6 levels in sera, and thus their data could not be determined via ELISA. Those data were eliminated from the analysis. We did not exclude the data of cytokine levels of subjects with antipyretic medication.

### Statistical analyses

Data were analyzed using unpaired Student’s *t*-test when comparing two experimental groups. Multiple comparisons were conducted using one-way analysis of variance (ANOVA) followed by the Tukey–Kramer post hoc test. Pearson correlation coefficients, and multiple-regression analysis were conducted using Prism v.7 or v.9 for Mac OS X. Error bars represent the standard deviation (SD). *P*-values less than 0.05 (*) were considered statistically significant. Statistical significance of the correlation coefficient of each pair of symptom scores (*p*-values) was calculated using Prism 7 software.

### Reporting summary

Further information on research design is available in the Nature Research Reporting Summary linked to this article.

## Supplementary information

Supplementary Information

REPORTING SUMMARY

## Data Availability

All data generated or analyzed during this study are included in the publication article and its supplementary information files.
