# Supplementary material for: Circulating extracellular vesicle microRNAs associated with adverse reactions, proinflammatory cytokine, and antibody production after COVID-19 vaccination
Source: NPJ Vaccines. 2022 Feb 8;7:16. doi: 10.1038/s41541-022-00439-3 (PMC8826357; doi:10.1038/s41541-022-00439-3)
Supplement: Supplementary file 1 — Supplementary Information [file 41541_2022_439_MOESM1_ESM.pdf]

Supplementary information

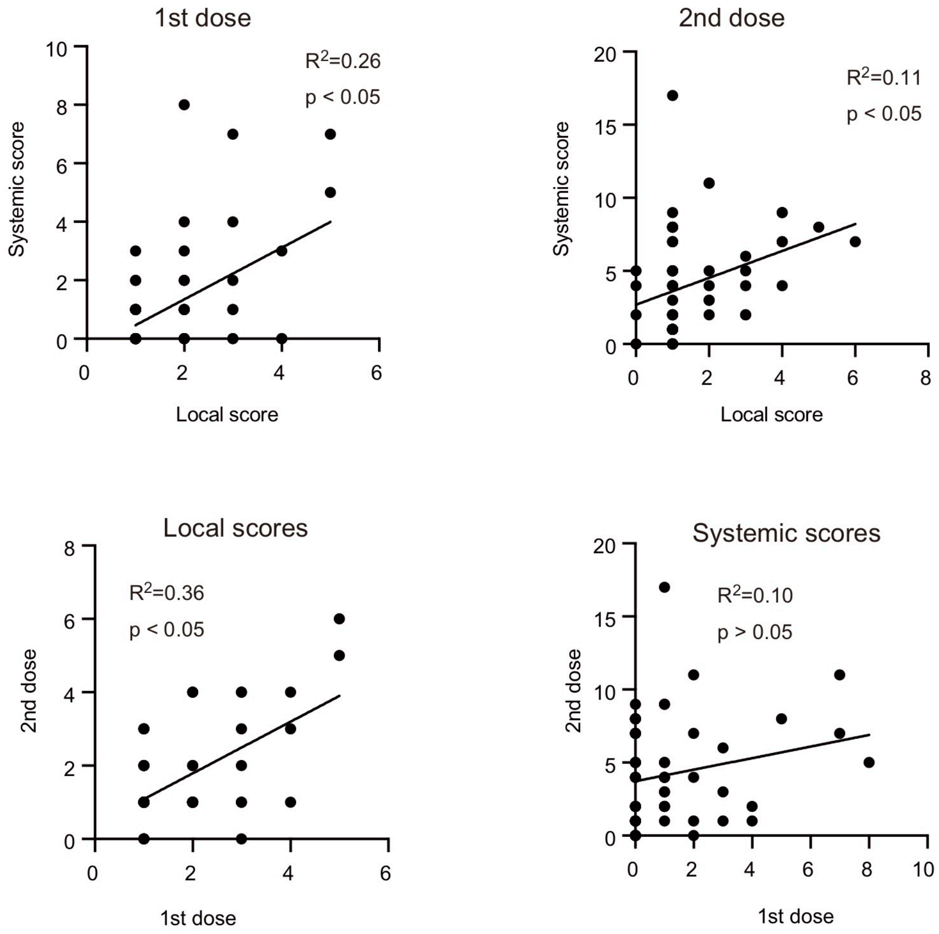

Supplementary Figure 1

Correlations among local and systemic scores after 1st and 2nd doses were investigated. Coefficient of determination (R^2) and p-values were calculated (Pearson correlation test). Each dot represents a subject.

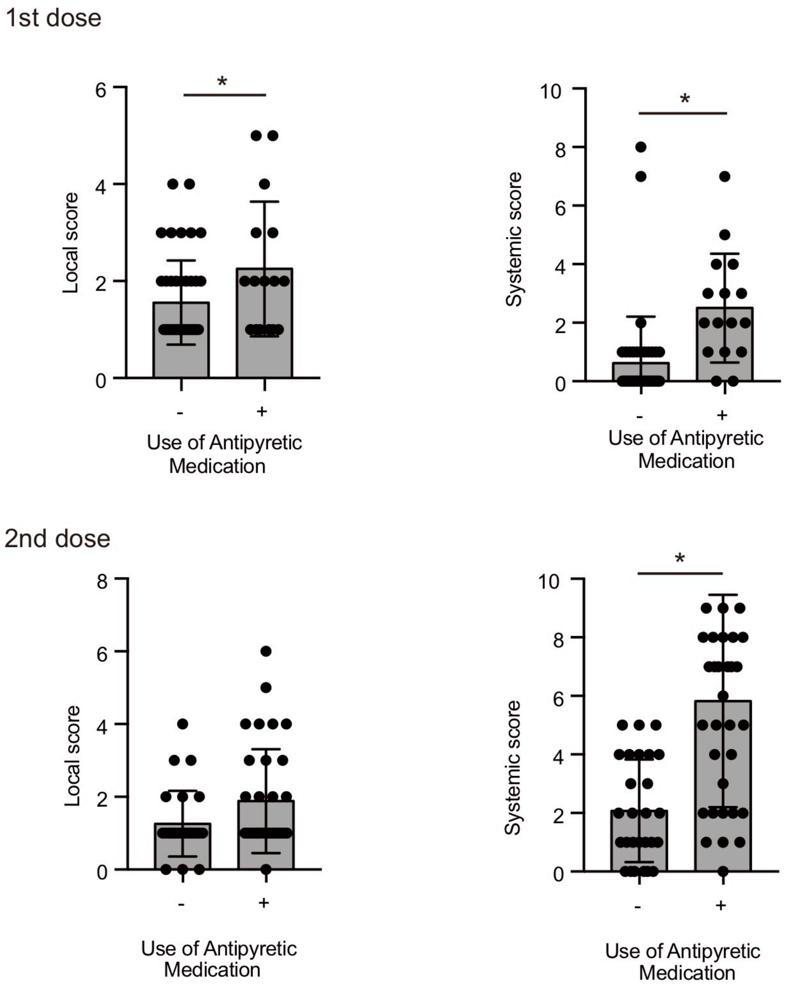

Supplementary Figure 2

Local and systemic scores of subjects with or without antipyretic medication were plotted. Each dot represents a subject. Student t-test were performed (*$p < 0.05$). The bars represent mean ± SD.

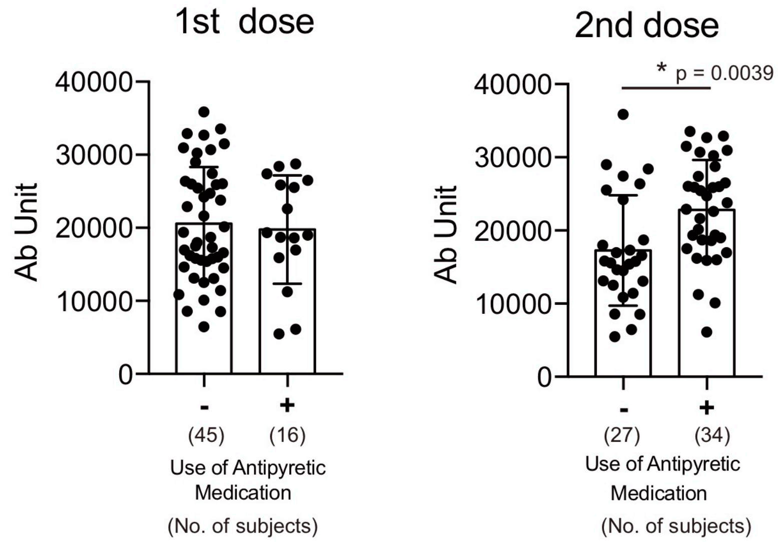

Supplementary Figure 3

Specific antibody titers of subjects with or without antipyretic medications were plotted. Each dot represents a subject. Student t-test were performed (*$p < 0.05$). The bars represent mean ± SD.

a

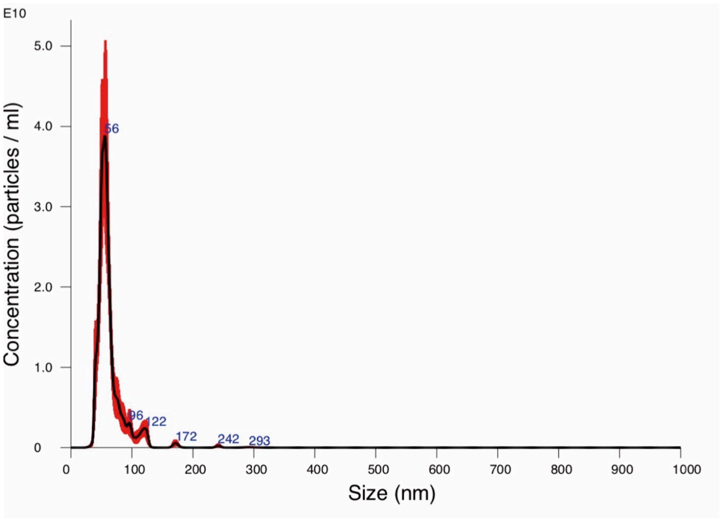

b

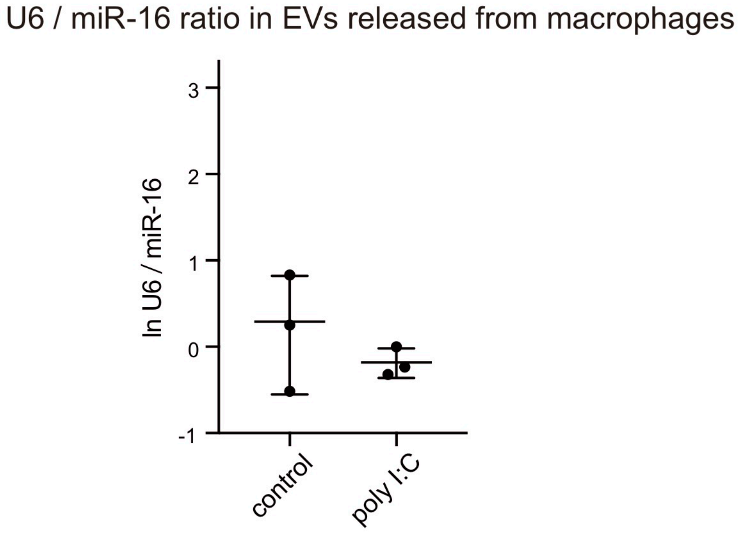

Supplementary Figure 4

a) Nanoparticle tracking analysis of EVs isolated from sera. The graph showed concentrations of particles at each size. An EVs samples were measured five times using NanoSight LM10V-HS. The data were analyzed with NTA3.2 software.

b) THP-1 macrophages were stimulated with poly I:C for 24 hours. EVs were collected from supernatants of macrophage cultures, and total RNAs were extracted. U6 and miR-16 RNA levels were determined by RT-qPCR. The bars represent mean ± SD.

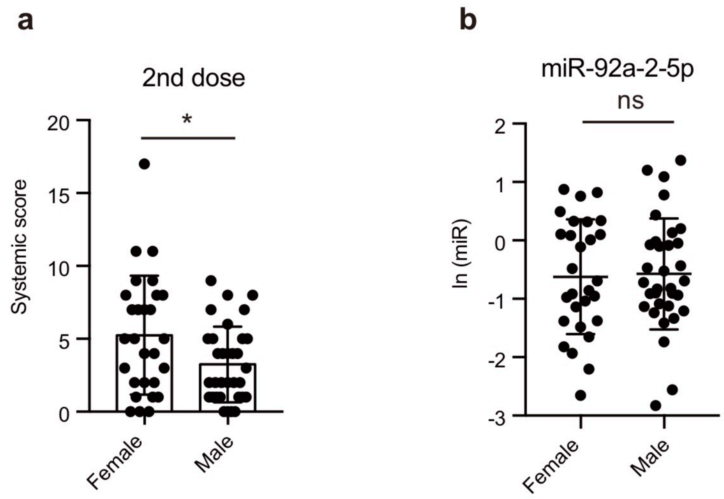

Supplementary Figure 5

a) Systemic scores after the second dose in female and male (*p < 0.05, t-test)

b) ln (miR-92a-2-5p) levels in female and male (ns: not significant, p > 0.05, t-test).

The bars represent mean ± SD.

1st dose	Swelling					
Local pain	none	mild	moderate	severe	grade 4	total
≦ mild	36 (90.0 %)	4 (10.0 %)	0 (0.0 %)	0 (0.0 %)	0 (0.0 %)	40
≧ moderate	11 (52.4%)	8 (38.1 %)	2 (9.5 %)	0 (0.0 %)	0 (0.0 %)	21

No. (%)

1st dose	Redness					
Local pain	none	mild	moderate	severe	grade 4	total
≦ mild	38 (95.0 %)	2 (5.0 %)	0 (0.0 %)	0 (0.0 %)	0 (0.0 %)	40
≧ moderate	17 (81.0 %)	3 (14.3 %)	1 (4.8%)	0 (0.0 %)	0 (0.0 %)	21

No. (%)

1st dose	Fatigue					
Local pain	none	mild	moderate	severe	grade 4	total
≦ mild	33 (82.5 %)	7 (17.5 %)	0 (0.0 %)	0 (0.0 %)	0 (0.0 %)	40
≧ moderate	10 (47.6 %)	6 (28.6%)	5 (23.8 %)	0 (0.0 %)	0 (0.0 %)	21

No. (%)

1st dose	Headache					
Local pain	none	mild	moderate	severe	grade 4	total
≦ mild	37 (92.5 %)	2 (5.0 %)	1 (2.5 %)	0 (0.0 %)	0 (0.0 %)	40
≧ moderate	15 (71.4%)	2 (5.0 %)	3 (14.3 %)	1 (4.8 %)	0 (0.0 %)	21

No. (%)

1st dose	Muscle pain					
Local pain	none	mild	moderate	severe	grade 4	total
≦ mild	33 (82.5 %)	7 (17.5 %)	0 (0.0 %)	0 (0.0 %)	0 (0.0 %)	40
≧ moderate	12 (57.1 %)	4 (19.0%)	5 (23. 8 %)	0 (0.0 %)	0 (0.0 %)	21

No. (%)

1st dose	Joint pain					
Local pain	none	mild	moderate	severe	grade 4	total
≦ mild	39 (97. 5%)	1 (2.5 %)	0 (0.0 %)	0 (0.0 %)	0 (0.0 %)	40
≧ moderate	16 (76. 2 %)	4 (19.0 %)	1 (4. 8 %)	0 (0.0 %)	0 (0.0 %)	21

No. (%)

2nd dose	Swelling					
Local pain	none	mild	moderate	severe	grade 4	total
≦ mild	42 (87.5 %)	6 (12.5 %)	0 (0.0 %)	0 (0.0 %)	0 (0.0 %)	48
≧ moderate	3 (23. 1 %)	6 (46.2 %)	4 (30.8 %)	0 (0.0 %)	0 (0.0 %)	13

No. (%)

2nd dose	Fever					
Local pain	none	mild	moderate	severe	grade 4	total
≦ mild	35 (72.9 %)	5 (10. 4 %)	6 (12. 5 %)	2 (4.2 %)	0 (0.0 %)	40
≧ moderate	9 (69. 2 %)	2 (15.4 %)	2 (15. 4 %)	0 (0.0 %)	0 (0.0 %)	21

No. (%)

2nd dose	Muscle pain					
Local pain	none	mild	moderate	severe	grade 4	total
≦ mild	27 (56.3 %)	17 (35.4 %)	3 (6.3 %)	1 (2.1 %)	0 (0.0 %)	40
≧ moderate	7 (53. 8 %)	3 (23.1 %)	2 (15.4 %)	1 (7.7 %)	0 (0.0 %)	21

No. (%)

Supplementary Table 1. % of adverse reactions

Supplementary Table 2. Primer list

miR-451a	AAACCGTTACCATTACTGAGTT
miR-21	TAGCTTATCAGACTGATGTTGA
miR-92a-2-5p	GGGTGGGGATTTGTTGCATTAC
miR-22	AAGCTGCCAGTTGAAGAACTGT
miR-23b	ATCACATTGCCAGGGATTACC
miR-30b	TGTAAACATCCTACACTCAGCT
miR-29a	TAGCACCATCTGAAATCGGTTA
miR-34a	TGGCAGTGTCTTAGCTGGTTGT
miR-107	AGCAGCATTGTACAGGGCTATCA
miR-148a	TCAGTGCACTACAGAACTTTGT
miR-148b	TCAGTGCATCACAGAACTTTGT
miR-221	AGCTACATTGTCTGCTGGGTTTC
miR-192	CTGACCTATGAATTGACAGCC
miR-16	TAGCAGCACGTAAATATTGGCG
miR-126-3p	TCGTACCGTGAGTAATAATGCG
miR-132-3p	TAACAGTCTACAGCCATGGTCG
miR-625-3p	GACTATAGAACTTTCCCCCTCA
