# Supplementary material for: Circulating extracellular vesicle microRNAs associated with adverse reactions, proinflammatory cytokine, and antibody production after COVID-19 vaccination
Source: NPJ Vaccines. 2022 Feb 8;7:16. doi: 10.1038/s41541-022-00439-3 (PMC8826357; doi:10.1038/s41541-022-00439-3)
Supplement: Supplementary file 2 — REPORTING SUMMARY [file 41541_2022_439_MOESM2_ESM.pdf]

nature portfolio

Corresponding author(s): Hiroyuki Oshiumi

Last updated by author(s): Nov 3, 2021

Reporting Summary

Nature Portfolio wishes to improve the reproducibility of the work that we publish. This form provides structure for consistency and transparency in reporting. For further information on Nature Portfolio policies, see our Editorial Policies and the Editorial Policy Checklist.

Statistics

For all statistical analyses, confirm that the following items are present in the figure legend, table legend, main text, or Methods section.

n/a	Confirmed	
☐	☒	The exact sample size (n) for each experimental group/condition, given as a discrete number and unit of measurement
☐	☒	A statement on whether measurements were taken from distinct samples or whether the same sample was measured repeatedly
☐	☒	The statistical test(s) used AND whether they are one- or two-sided<br>*Only common tests should be described solely by name; describe more complex techniques in the Methods section.*
☐	☒	A description of all covariates tested
☐	☒	A description of any assumptions or corrections, such as tests of normality and adjustment for multiple comparisons
☐	☒	A full description of the statistical parameters including central tendency (e.g. means) or other basic estimates (e.g. regression coefficient) AND variation (e.g. standard deviation) or associated estimates of uncertainty (e.g. confidence intervals)
☐	☒	For null hypothesis testing, the test statistic (e.g. F, t, r) with confidence intervals, effect sizes, degrees of freedom and P value noted<br>*Give P values as exact values whenever suitable.*
☒	☐	For Bayesian analysis, information on the choice of priors and Markov chain Monte Carlo settings
☒	☐	For hierarchical and complex designs, identification of the appropriate level for tests and full reporting of outcomes
☒	☐	Estimates of effect sizes (e.g. Cohen's d, Pearson's r), indicating how they were calculated

Our web collection on statistics for biologists contains articles on many of the points above.

Software and code

Policy information about availability of computer code

Data collection	StepOne and StepOnePlus Software v2.3
Data analysis	GraphPad Prism version 9.2.9, Microsoft Excel for Mac 16.16.21.

For manuscripts utilizing custom algorithms or software that are central to the research but not yet described in published literature, software must be made available to editors and reviewers. We strongly encourage code deposition in a community repository (e.g. GitHub). See the Nature Portfolio guidelines for submitting code & software for further information.

Data

Policy information about availability of data

All manuscripts must include a data availability statement. This statement should provide the following information, where applicable:

- Accession codes, unique identifiers, or web links for publicly available datasets
- A description of any restrictions on data availability
- For clinical datasets or third party data, please ensure that the statement adheres to our policy

All data generated or analyzed during this study are included in the publication article and its supplementary information files.

March 2021

Field-specific reporting

Please select the one below that is the best fit for your research. If you are not sure, read the appropriate sections before making your selection.

☒ Life sciences ☐ Behavioural & social sciences ☐ Ecological, evolutionary & environmental sciences

For a reference copy of the document with all sections, see nature.com/documents/nr-reporting-summary-flat.pdf

Life sciences study design

All studies must disclose on these points even when the disclosure is negative.

Sample size	The sample size was estimated based on previous studies (ref 14 and 15), and was determined to be adequate on the basis on the magnitude and consistency of measurable differences between groups.
Data exclusions	Basal serum IL-6 levels of a few subjects were too high to measure its concentration by ELISA, and thus those data were excluded (see materials and methods). No other data were excluded.
Replication	qPCR was performed with technical duplicate samples.
Randomization	Participants were recruited to this study based on their agreements. We did not recruit the peoples whose medical conditions were not stable.
Blinding	The authors who collected the samples and data were different from those who analyzed the data.

Reporting for specific materials, systems and methods

We require information from authors about some types of materials, experimental systems and methods used in many studies. Here, indicate whether each material, system or method listed is relevant to your study. If you are not sure if a list item applies to your research, read the appropriate section before selecting a response.

Materials & experimental systems

n/a	Involved in the study	
☒	☐	Antibodies
☐	☒	Eukaryotic cell lines
☒	☐	Palaeontology and archaeology
☒	☐	Animals and other organisms
☐	☒	Human research participants
☒	☐	Clinical data
☒	☐	Dual use research of concern

Methods

n/a	Involved in the study	
☒	☐	ChIP-seq
☒	☐	Flow cytometry
☒	☐	MRI-based neuroimaging

Eukaryotic cell lines

Policy information about cell lines

Cell line source(s)	THP-1 cells
Authentication	The cell line was not authenticated. We obtained TH`-1 cells from JCRB cell bank.
Mycoplasma contamination	We have confirmed that there are no mycoplasma contamination using a mycoplasma detection kt.
Commonly misidentified lines (See ICLAC register)	No commonly misidentified cell lines were used in this study.

Human research participants

Policy information about studies involving human research participants

Population characteristics	Population characteristics is described in Fig 1c.
Recruitment	Between March, 2021 and June, 2021, 61 participants mainly including the staff and faculty members in Kumamoto University and its hospital were recruited. Those with unstable disease conditions or symptoms were not recruited to this study. All participants provided written informed consent.

Ethics oversight

The ethics committee of the Faculty of Life Science at Kumamoto University approved this study (RINRI 1524), and all experiments have been conducted according to the principles expressed in the Declaration of Helsinki.

Note that full information on the approval of the study protocol must also be provided in the manuscript.
